# Commentary: Blood-Derived microRNAs for Pancreatic Cancer Diagnosis: A Narrative Review and Meta-Analysis

**DOI:** 10.3389/fphys.2018.01896

**Published:** 2019-01-22

**Authors:** Rama Jayaraj, Chellan Kumarasamy, Shanthi Sabarimurugan, Siddhartha Baxi

**Affiliations:** ^1^College of Health and Human Sciences, Charles Darwin University, Darwin, NT, Australia; ^2^School of Health, Charles Darwin University, Darwin, NT, Australia; ^3^School of Public Health, University of Adelaide, Adelaide, SA, Australia; ^4^School of Bioscience and Technology, VIT University, Vellore, India; ^5^Genesis Cancer Care Centre, Bunbury, WA, Australia

**Keywords:** miRNAs, meta-analysis, diagnosis, pancreatic cancer, narrative review

Liu et al. have recently published a paper highlighting blood-derived miRNAs as possible early diagnostic biomarkers in Pancreatic Cancer by conducting a narrative review and meta-analysis titled, “Blood-Derived microRNAs for Pancreatic Cancer Diagnosis: A Narrative Review and Meta-Analysis” (Li et al., 2018). Considering the extreme aggressiveness and malignancy associated with Pancreatic Cancer, combined with its poor prognosis and survival rates, and the dependence of diagnosis on non-specific tumor biomarkers, Liu et al.'s study highlighting possible alternative biomarkers, is definitely one that is of great clinical value (Benzel and Fendrich, 2018). However there are a few issues with this study that may serve to hinder its practical clinical applicability. First and foremost, in fairness we would like to highlight that Liu and associates are not the first to attempt to determine the applicability of miRNAs as diagnostic biomarkers in Pancreatic Cancer, multiple such meta-analysis studies have been conducted and published in 2012, 2014, 2017 (and have not been cited by the authors of this study) and even in May of 2018 (Wan et al., 2012; Ding et al., 2014; Pei et al., 2017; Sun et al., 2018).

Despite this, Liu et al.'s study stands out by the virtue of being the most updated study regarding this subject, and is therefore expected to have the largest pool of clinical data to support its meta-analysis. However, the study published by Sun et al. in May 2018 in the journal Disease Markers, titled, “Systematic Review and Meta-Analysis of Diagnostic Accuracy of miRNAs in Patients with Pancreatic Cancer,” includes a pool of 40 studies for its meta-analysis when compared to Liu et al.'s 17 studies (Sun et al., 2018). This is in particular, an issue when we consider that Sun et al.'s search was up to June 30, 2016 and Liu et al.'s search was up to February 1, 2018, and that the search was conducted in the same set of bibliographic databases using similar search terms. The value and quality of any systematic review and meta-analysis study is dependent on the pool of research data included in the study, and an updated meta-analysis paper evaluating a smaller pool of research data when compared to older studies, severely hampers its utility and calls into question its clinical viability.

Though, it is worth highlighting, that the results of Liu et al.'s study conform to the results presented in aforementioned, previously published papers. All previous studies unanimously declare that the sensitivity and specificity of multiple miRNA profiles are superior to that of single miRNA profiles for pancreatic cancer diagnosis. However, a few single miRNA have been suggested to be more valuable as indicators of pancreatic cancer, compared to other miRNA. Li et al, Sun et al, Wan et al, and Pei et al.'s studies, all describe miR-21 as a promising marker for pancreatic cancer (Wan et al., 2012; Pei et al., 2017; Li et al., 2018; Sun et al., 2018). Pei et al.'s study goes on to highlight miR-155 and miR-210 as other miRNAs that feature prominently in clinical miRNA profile analysis, while Wan et al. list more than 20 miRNA that were analysed in their study, including miR-16, 20a, 24, 25, 99a, 100, 135b, 125b, 155, 181a, 185, 191, 196a, 210, 212, 221, 301, 367a, and let-7i (Wan et al., 2012; Pei et al., 2017). Ding et al.'s study on the other hand does not specify any singular miRNA, and limits itself to pooled analysis of sensitivity and specificity (Ding et al., 2014).

Additionally, we would like to note that previous studies evaluating diagnostic test accuracy have shown that Chi-square and I-square values alone for analysis of pooled meta-analysis data may not be sufficiently informative as they do not consider threshold effect. In random effects model of meta-analysis, Tau-square value may also be included as the estimated variation or heterogeneity between the effects for test accuracy observed in different studies (Lee et al., 2015).

Finally, considering the current evidence presented by Li et al., and previous studies, it is clear that miRNAs have potential clinical utility as diagnostic and prognostic markers in Pancreatic Cancer. Although Li et al., study presents potentially interesting data and results, the highlighted issues need to be resolved before it may be considered robust enough a study to be cited by and used for future research in this field.

## Author Contributions

RJ conceived of this critical review and led the development of the letter to the editor. RJ and CK wrote the first draft of the letter, and coordinated and integrated comments from co-authors. SS, and SB critically revised and edited successive drafts of the manuscript. All authors read and approved the final version of the manuscript.

### Conflict of Interest Statement

The authors declare that the research was conducted in the absence of any commercial or financial relationships that could be construed as a potential conflict of interest.
